# Rapid and Simultaneous Detection of Petroleum Hydrocarbons and Organic Pesticides in Soil Based on Electronic Nose

**DOI:** 10.3390/s25020380

**Published:** 2025-01-10

**Authors:** Cheng Kong, Lin Sun, Xiaodan Li, Yu Yan, Zhiyong Chang, Mo Li, Fuyan Gou, Baojun Rong

**Affiliations:** 1 College of Biological and Agricultural Engineering, Jilin University, Changchun 130022, China; kongcheng20@mails.jlu.edu.cn (C.K.); zychang@jlu.edu.cn (Z.C.); 2 Beijing Institute of Geohazard Prevention and Control, Beijing 100011, China; dr.l.sun@petalmail.com; 3 China Northeast Municipal Engineering Design and Research Institute Co., Ltd., Changchun 130021, China; 1360432@163.com (X.L.); 18686670076@163.com (Y.Y.); 4 School of Engineering and Technology, China University of Geosciences, Beijing 100083, China; 5 National Key Laboratory of Automotive Chassis Integration and Bionics, Jilin University, Changchun 130022, China; 6 College of Geoexploration Science and Technology, Jilin University, Changchun 130022, China; 7 School of Mechanical and Vehicle Engineering, Jilin Engineering Normal University, Changchun 130052, China

**Keywords:** soil rapid detection, petroleum hydrocarbon pollution, organic pesticide pollution, electronic nose, recognition model

## Abstract

The rapid detection of petroleum hydrocarbons and organic pesticides is an important prerequisite for precise soil management. It is also a guarantee for soil quality, environmental safety, and human health. However, the current rapid detection methods are prone to sample matrix interference, complex development processes, short lifespan, and low detection accuracy. Moreover, they face difficulties in achieving simultaneous detection of petroleum hydrocarbons and organic pesticides. In this paper, we developed an electronic nose system for the simultaneous detection of petroleum hydrocarbons and organic pesticides in soil based on gas technology, which includes a sampling module and recognition model. The developed sampling module can simultaneously acquire the odor signals of petroleum hydrocarbons and organic pesticides in soil. The established recognition model can quickly distinguish between healthy soil, soil contaminated by petroleum hydrocarbons, and soil contaminated by organic pesticides. It can also achieve specific recognition of pesticide types and petroleum types. The performance of the developed electronic nose system was verified for real soil, petroleum products, and organic pesticides. The experiment shows that the developed electronic nose system has an accuracy of 100% for three tasks: soil conditions identification, pesticide types identification, and petroleum types identification.

## 1. Introduction

With the rapid development of modern industry and agriculture, petroleum hydrocarbons and organic pesticides have become typical organic pollutants that endanger soil health. Petroleum hydrocarbons and organic pesticides in the soil continue to cause a variety of pollution hazards [1]. On the one hand, they can lead to serious consequences for soil such as compaction, acidification, nutrient loss, and inhibition of microbial activity [2,3]. On the other hand, they can pollute the atmospheric environment through volatilization, and pollute water through leaching percolation, leading to continuous deterioration of the overall ecological environment [4,5]. More importantly, petroleum hydrocarbons and organic pesticides in soil can accumulate through the food chain and harm human health, leading to serious diseases such as infertility, Alzheimer’s, neonatal malformations, and cancer [6,7,8]. In addition, petroleum hydrocarbons and organic pesticides are important sources of other organic pollutants such as polycyclic aromatic hydrocarbons and benzene compounds, whose presence further complicates the complexity of soil organic pollution. Effective detection of petroleum hydrocarbons and organic pesticides can nip pollution hazards in the bud and guide the precise treatment of contaminated soil.

The conventional detection methods for petroleum hydrocarbons and organic pesticides in soil are mainly based on chromatography and chromatography coupling technology. They have the advantages of high selectivity, high sensitivity, and high precision. They have become the standard methods for detecting petroleum hydrocarbons and organic pesticides in soil [9,10,11]. But their detection costs are high, detection time is long, equipment volume is large, and operation is complex, making it impossible to achieve rapid in situ detection. Conventional detection methods are increasingly unable to meet modern agricultural detecting needs. In response to the demand for rapid detection of soil pollution, researchers have developed rapid detection methods for petroleum hydrocarbons based on optical technology [12,13] and organic pesticides based on biotechnology, spectroscopy [14,15,16]. However, these rapid detection methods still have drawbacks such as susceptibility to sample matrix interference, complex development process, short lifespan, and low detection accuracy. More importantly, these methods face difficulties in achieving simultaneous detection of petroleum hydrocarbons and organic pesticides in soil.

Both petroleum hydrocarbons and organic pesticides produce volatile organic compounds. Gas sensing technology is expected to achieve simultaneous detection of petroleum hydrocarbons and organic pesticides in soil. As a typical multi-component gas sensing system, electronic nose (E-nose) technology has the advantages of low cost, fast detection speed, and simple operation. It has become an important research direction for rapid in situ detection of complex gases [17,18,19,20]. At present, researchers have used E-nose to independently detect petroleum hydrocarbons and organic pesticides in soil, verifying their detection effectiveness [21,22]. For example, Qiao et al. [23] proposed a hybrid model based on unsupervised and supervised learning for detecting pesticides in soil. Chakraborty K et al. [24] elaborated on the advantages and applications of E-nose in pesticide detection. Shi et al. [25] successfully proposed a two-stage framework to identify the categories and concentrations of pesticide residues. Yu et al. [26] elaborated on the detection of pesticide residues using rapid detection technologies such as E-nose. Currently, these studies only focus on developing E-nose systems or researching identification algorithms for a specific type of pollution, such as individual pesticide pollution or individual petroleum pollution. There are few reports on developing an E-nose system for simultaneous detection of petroleum hydrocarbons and organic pesticides in soil.

In this study, we developed an integrated E-nose system for the simultaneous detection of petroleum hydrocarbons and organic pesticides in soil, which includes a sampling module and a recognition model. The sampling module can simultaneously obtain odor signals of petroleum hydrocarbons and organic pesticides in soil, with fast sampling speed, small volume, and low cost. The recognition model can quickly distinguish between healthy soil, petroleum hydrocarbons contaminated soil, and organic pesticides contaminated soil. It can also further achieve specific recognition of each pollutant. The detection results for real soil, petroleum products, and organic pesticides show that the developed E-nose system can effectively achieve simultaneous detection of petroleum hydrocarbons and organic pesticides in soil.

## 2. Materials and Methods

### 2.1. Real Soil, Petroleum Products, and Organic Pesticides

The soil samples used for pollution detection experiments in this paper were collected from Panshi City, Jilin Province (42° N, 126° E). The spacing between soil sampling points was greater than 2 m, and a steel shovel was used to collect soil layers with a depth of 0–20 cm. The soil was air-dried at room temperature (24 °C) for 7 days after collection. Then the soil was sieved through a 2 mm screen to remove larger particles and mixed evenly. The processed soil was confirmed by gas chromatography to be free of petroleum hydrocarbons and any organic pesticides.

Diesel and gasoline are widely used fuels in agricultural machinery and transportation. The # 92 gasoline and # 0 diesel purchased from gas stations in Changchun, Jilin Province were used as sources of petroleum hydrocarbon pollution. In order to demonstrate the representativeness of organic pesticides, taking into account pesticide types, usage amounts, application functions, and pollution hazards, chlorpyrifos, cyfluthrin, deltamethrin, dithane, glyphosate, and mancozeb were selected as sources of organic pesticide pollution. The selected organic pesticides were purchased from the pesticide market in Changchun, Jilin Province.

**Figure 1:**
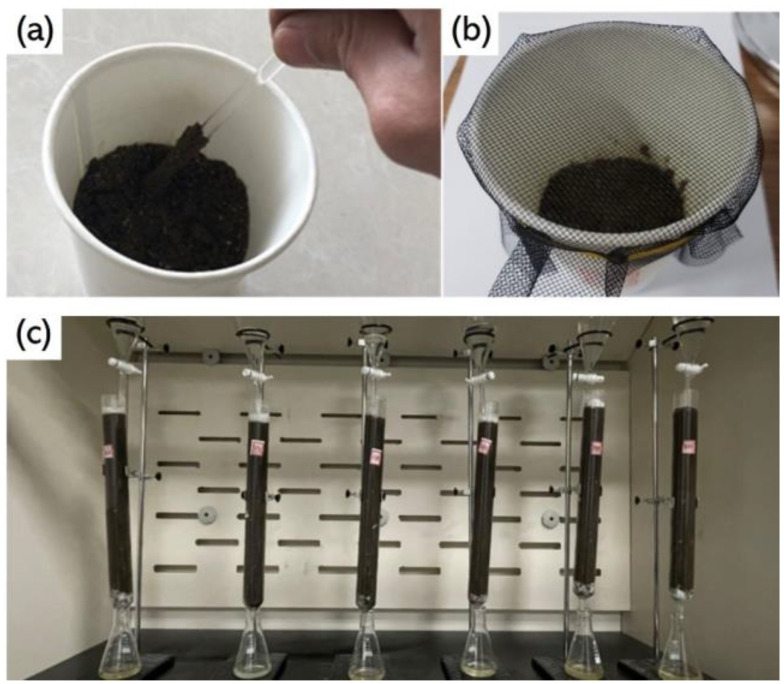
Preparation of polluted soil: (**a**) Diesel and water titration stirring (**b**) Dust prevention treatment (**c**) Leaching experiment of organic pesticides.

In order to obtain a large number of soil samples contaminated with different types of petroleum hydrocarbons and organic pesticides, artificial simulation methods were used to produce soil pollution samples. Following our previous research plan [27], the preparation process of soil samples contaminated with petroleum hydrocarbons is shown in Figure 1a,b. Firstly, put 100 g of air-dried and evenly mixed soil into a glass cup, then add 15 g of water and stir until the relative humidity of the soil is 80%. Finally, use a pipette to titrate the soil in the glass cup at a pollutant concentration of 3–5%. In order to reduce experimental errors and ensure objectivity, 120 samples were prepared from soil contaminated with # 92 gasoline and 0 # diesel, for a total of 240 samples. Unlike petroleum hydrocarbons, which are mainly concentrated in the top 20 cm of soil, organic pesticides have stronger migration behavior and pollute deeper soil layers. Therefore, in order to better simulate the migration and pollution of organic pesticides in soil, a soil leaching experiment was conducted to prepare soil samples contaminated with organic pesticides [28], as shown in Figure 1c. Among them, 40 samples were taken from soil contaminated with each pesticide, and 240 samples were taken from healthy soil.

### 2.2. Construction of Sampling Module for Simultaneous Detection of Soil Pollution

**Figure 2:**
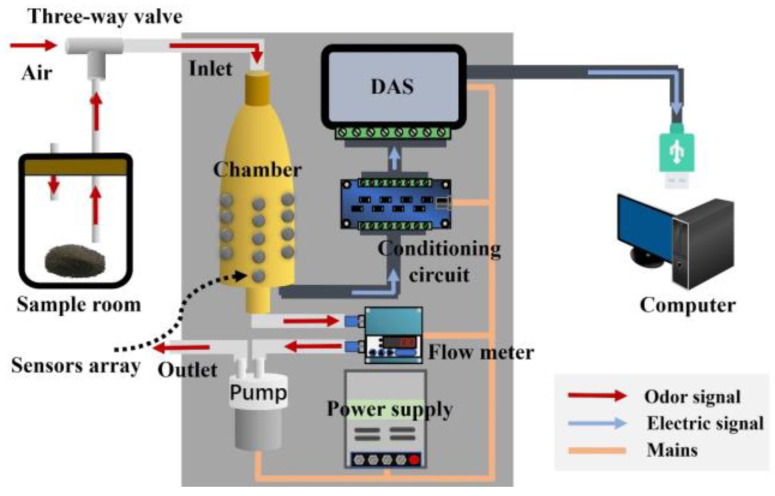
Design scheme of the sampling module for the E-nose system.

The overall design scheme of the sampling module for soil odor signal acquisition is shown in Figure 2. The sampling module is based on a gas array, with the sensor chamber as the key component for efficient sampling. The entire system includes a gas generation unit, detection unit, data acquisition unit, and power supply. The gas generation unit mainly relies on the sample room to work, producing enough volatile gas from soil samples for detection through a dynamic headspace during the process. The detection unit mainly uses Teflon tubes, electromagnetic valves, a gas flow meter, a miniature air pump, and a gas sensors array to convert the odors into electrical signals. The conditioning circuit and data acquisition system (DAS) collect, filter, amplify, and analog-to-digital (AD) convert the electrical signals generated by the gas, generating digital signals that can be used for subsequent analysis and pattern recognition. Among them, the DAS uses a USB5631 data acquisition card (ART Technology, Beijing, China), which can read signals from 26 sensors at high speed. The power supply mainly powers the entire sampling module. The numerical signals obtained by the sampling module ultimately enter the data analysis module, which can be either a host computer or an integrated chip. For efficient data processing and model verification, a host computer was chosen as the data analysis module in this paper.

The sensitivity and detection range of gas sensors determine the upper limit of the E-nose system. Selecting appropriate and effective sensors is crucial for gas detection. Metal oxide semiconductor (MOS) sensors are well-suited for large-scale deployment and rapid detection in agricultural environments due to their low cost, ease of integration, high sensitivity, and fast response and recovery times [29]. Meanwhile, taking into account that the acquisition module needs to detect many volatile odors in petroleum hydrocarbons and organic pesticides, 26 MOS sensors were selected to form the sensors array. These sensors are sensitive to most volatile organic compounds and can enhance the detection sensitivity of E-noses for petroleum hydrocarbons and organic pesticides. Detailed information about the sensors used is shown in Appendix A.

**Figure 3:**
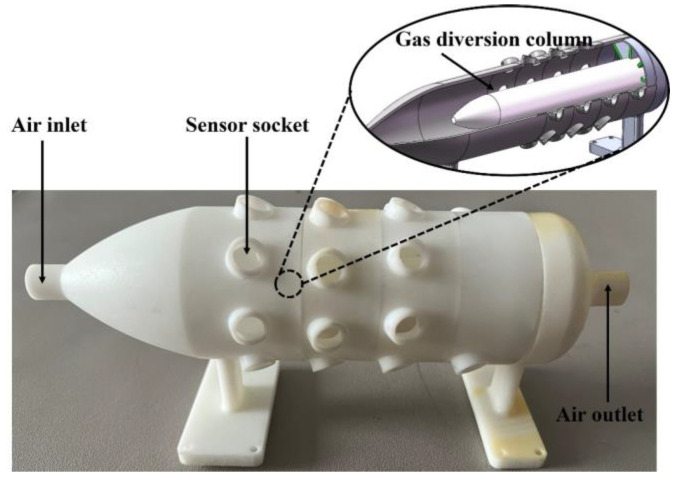
Sensor chamber in the sampling module.

The sensor chamber employs a substantially cylindrical chamber, as shown in Figure 3. The chamber has a small opening and a larger chamber body, a transition region set between the inlet and the sensor array, allowing the high-speed airflow at the inlet to flow as uniformly as possible into the sensor area after entering the chamber. There is a diversion column near the sensor sockets, which diverts more airflow to the vicinity of the sensors’ surfaces, enhancing signal responses. The socket arrays are distributed in a circular pattern at a certain angle around the outermost part of the chamber cylinder, ensuring uniform airflow at each sensor and allowing the gas to pass through more smoothly.

**Figure 4:**
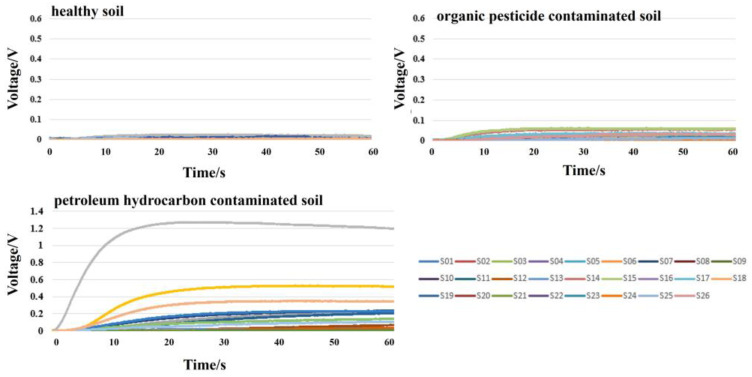
Representative response signal curves.

The sampling module was used to collect soil sample odor signals in a temperature and humidity-stable environment. During the odor signal acquisition process, the gas flow rate was set at 300 mL/min, the sampling time was 1 min, and the sampling frequency was 100 Hz. After the sampling of one sample, clean air was used to thoroughly wash the sampling module for 5 min. The representative response signal curves obtained are shown in Figure 4.

### 2.3. Construction of Recognition Model for Simultaneous Detection of Soil Pollution

Aiming at rapid monitoring of pollution and precise remediation of soil, a distributed soil pollution recognition model is proposed in this paper, with both accuracy and efficiency being considered. The recognition model mainly consists of three functional modules: soil condition detection, petroleum hydrocarbon-specific identification, and pesticide-specific identification. The recognition model composed of separately layered functional modules has the following advantages:

- The recognition model can simultaneously detect soil petroleum hydrocarbon pollution and organic pesticide pollution, fully reflecting the soil pollution status, and providing accurate support for soil rapid monitoring and precise remediation;
- Each individual functional module needs to process only a limited amount of data, greatly reducing the energy consumption required for data transmission, computation, and storage across the entire model, while improving detection efficiency;
- Each independent functional module only needs to identify a specific soil, making the detection more targeted and significantly improving the detection accuracy.

**Figure 5:**
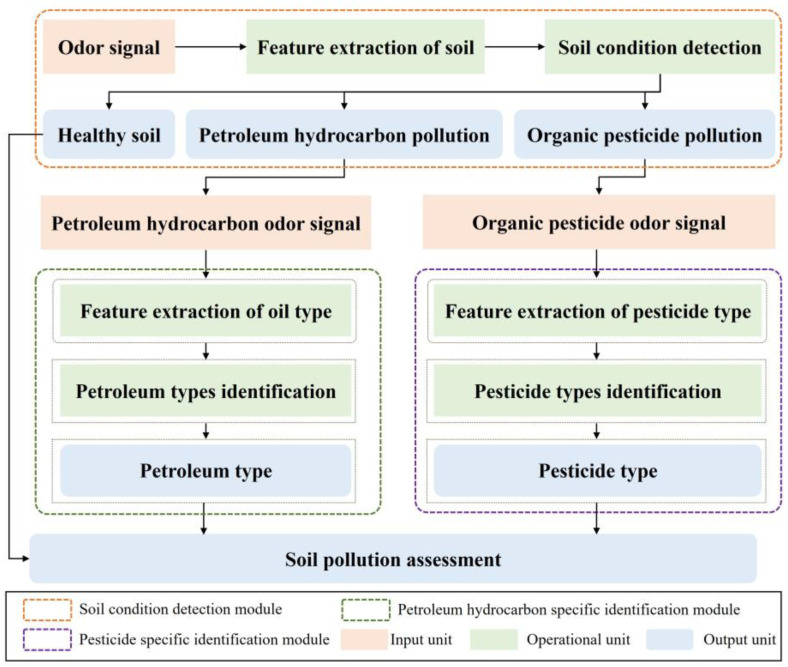
The framework of distributed soil pollution recognition model.

The proposed distributed soil pollution recognition model framework is shown in Figure 5. The goal of the soil condition detection module is to determine whether the soil is contaminated with petroleum hydrocarbons or organic pesticides and to identify the type of pollution. When the detection result is healthy soil, the detection ends. When the detection result is petroleum hydrocarbon pollution, the corresponding odor signal enters the petroleum hydrocarbon-specific identification module to achieve accurate identification of the petroleum type. When the detection result is organic pesticide pollution, the corresponding odor signal enters the organic pesticide-specific identification module to achieve accurate identification of pesticide type.

Different analysis tasks often require different feature extraction methods and classifiers. The choice of appropriate feature extraction methods and classifiers is crucial to the analysis results. During the process of soil detection and analysis, we selected the average (Ave), fast Fourier transform (FFT), integral value (IV), maximum (Max), polynomial curve fitting (PCF), and wavelet transform (WT) as feature extraction methods, which have shown good performance in previous studies [30]. Among them, Ave takes the average of the sensor response values during the whole process as a feature. FFT decomposes the original response into a superposition of direct current components and different harmonic components and transforms coefficients as response features. IV is the area enclosed by the response signal curve. Max takes the maximum value of the sensor response values during the whole process as a feature. PCF uses polynomial functions to fit response curves and takes the fitting coefficients as features. WT is an extension of FFT that maps signals into a new space, decomposes the original response into approximations (low frequency) and details (high frequency), and uses transform coefficients as features. In our previous independent research on soil petroleum hydrocarbons and soil pesticide detection, we compared numerous recognition methods. Among them, support vector machines (SVM) and random forests (RF) showed better performance [27,28,31]. Therefore, in this paper, we directly use these two methods to verify the performance of the developed E-nose system in detecting soil petroleum hydrocarbons and pesticides simultaneously. SVM and RF are widely used supervised learning algorithms for classification and regression analysis. Regarding the determination of machine learning parameters, we used the grid optimization method to determine the optimal parameters for each model. The optimization scope for each parameter is shown in Appendix A. The acquired dataset was analyzed using 10-fold cross-validation with different feature extraction methods and classifiers. 10-fold cross-validation divides the dataset into 10 independent parts, taking turns using 9 parts as the training set and 1 part as the testing set. The average of the 10 results is used as an estimate of the algorithm’s accuracy. This method ensures that the training and testing sets of each model do not have the same samples, effectively avoiding model overfitting and objectively evaluating algorithm performance. Finally, the feature extraction method and classifier with the highest average recognition rates were optimized for the most suitable soil pollution assessment.

## 3. Results and Discussion

### 3.1. Soil Condition Detection

**Figure 6:**
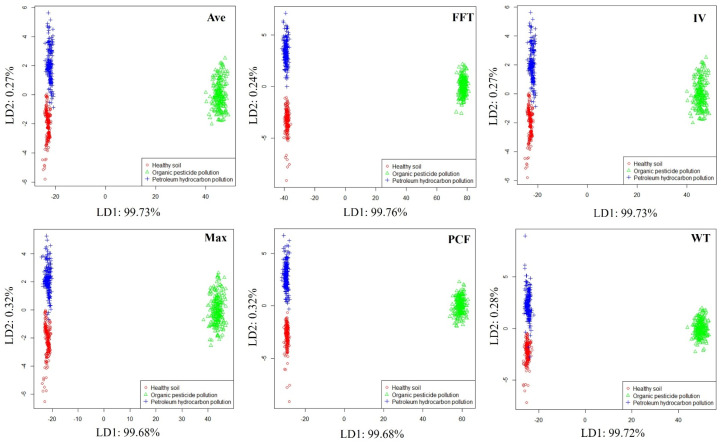
Distribution of samples from different soil conditions.

In order to preliminarily evaluate the ability of the E-nose system to distinguish different soil conditions, linear discriminant analysis (LDA) [32] was used to visualize the odor data of healthy soil, petroleum hydrocarbon-contaminated soil, and organic pesticide-contaminated soil. LDA can explore the distribution patterns of samples from different soil conditions in the feature space. The LDA visualization results obtained are shown in Figure 6, where soils in different conditions can be clearly distinguished in all feature spaces. Moreover, it can be detected if soil contaminated with organic pesticides is very far away from healthy soil and petroleum hydrocarbon-contaminated soil in the feature space, which may be related to the high volatile components and good odor volatility of organic pesticides.

**Figure 7:**
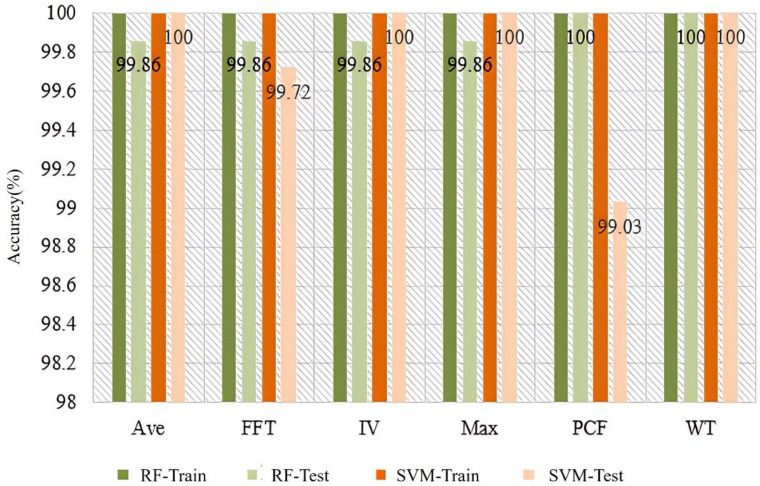
Soil condition detection based on different features and classifiers.

The results of soil detection in different conditions using Ave, FFT, IV, Max, PCF, and WT feature extraction methods, the SVM and RF classifiers are shown in Figure 7. According to the results, the accuracy of all combination schemes in the training set can reach 100%, while the accuracy in the testing set can also reach over 99%. This is mainly because the volatile compounds in healthy soil, petroleum hydrocarbon-contaminated soil, and organic pesticide-contaminated soil are significantly different, making it easy to distinguish them using odors. From the classifier’s recognition results, SVM has better classification performance, achieving an accuracy of 100% for healthy soil, petroleum hydrocarbon-contaminated soil, and organic pesticide-contaminated soil under various feature extraction methods. Among the various feature extraction methods, Ave, IV, Max, and WT all show good recognition performance in terms of accuracy, while PCF and FFT feature extraction methods are slightly weaker. This may be mainly due to the high dimensionality of PCF and FFT features, which leads to overfitting of the model [33], resulting in a decrease in the accuracy of the testing set.

### 3.2. Specific Identification of Petroleum Hydrocarbons and Organic Pesticides

**Figure 8:**
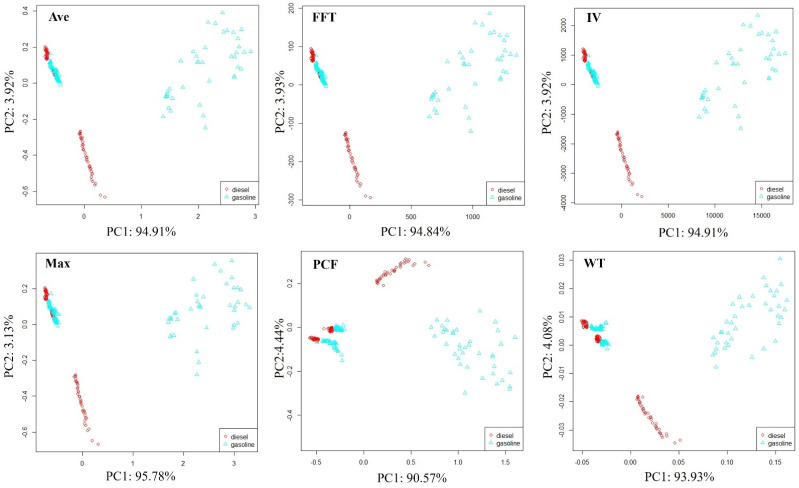
Distribution of soil samples polluted by diesel and gasoline.

The petroleum products used in this paper are only diesel and gasoline. Using LDA for data dimensionality reduction visualization cannot clearly represent their distribution patterns in the feature space. Therefore, in order to preliminarily evaluate the system’s ability to distinguish petroleum types, principal component analysis (PCA) [34] was used to reduce the dimensionality and visualize soil samples contaminated with different petroleum types l, as shown in Figure 8.

The distribution pattern of samples in all feature spaces tends to be consistent; samples can be divided into three parts. Some soil samples contaminated with diesel and gasoline can be clearly distinguished. Some soil samples contaminated with diesel and gasoline are also clustered together, and although they can be distinguished, the boundary for differentiation is not clear. The soil samples contaminated with diesel and gasoline clustered together were detected after 7 days of soil sample preparation. In comparison, when soil samples were just made, the volatile organic compounds emitted by samples after 7 days may be greatly reduced, which weakens the E-nose response signal and makes it difficult to distinguish them using simple PCA.

The results of modeling and predicting petroleum types using SVM and RF are shown in Table 1. The developed E-nose system can achieve specific identification of different petroleum types. Using the Max feature extraction method to extract petroleum-type features and using SVM for recognition, the highest accuracy can reach 100%. It can be found that Max feature values have better performance in extracting petroleum-type features, while RF classifiers have better overall performance in type recognition by comparison.

**Figure 9:**
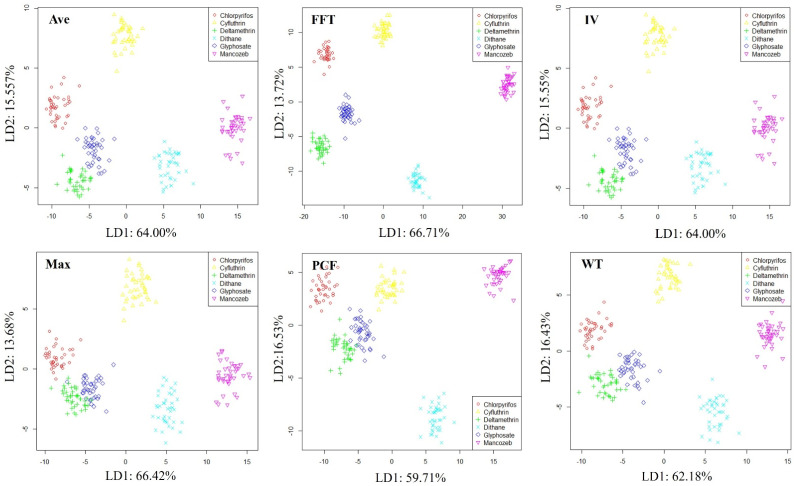
Distribution of soil samples polluted by different organic pesticide types.

In order to preliminarily evaluate the system’s ability to distinguish pesticide types, LDA was used to visualize soil samples contaminated with different pesticide types, as shown in Figure 9. The distribution pattern of samples in all feature spaces tends to be consistent, with all samples clustered into six parts, and the samples between each part can be clearly distinguished. According to our experimental records, these six parts correspond to six different pesticide types, indicating that the developed E-nose system has good performance in identifying pesticide types.

The results of predicting pesticide types using SVM and RF are shown in Table 2. From the table, it can be seen that the developed E-nose system can achieve specific identification of different pesticide types. Using the Max feature extraction method to extract pesticide-type features and using SVM for identification, the highest accuracy can reach 100%. In terms of feature extraction and classification methods, based on the average accuracy, it can be found that there is little difference in the results of pesticide-type identification between feature extraction methods and classifiers. Any feature extraction method and classifier can achieve good recognition performance.

The above results indicate that the developed E-nose system can simultaneously achieve rapid detection and specific identification of pollution types in soil. Compared with traditional chromatography, the E-nose system has significant advantages in development cost, equipment size, and detection time. The total volume of the sampling module is 8372 cm^3^ (28 cm × 23 cm × 13 cm), with a maximum power consumption of 15.1 W and a total cost of approximately USD 771. The entire detection time includes sampling and calculation. The sampling time is 60 s, while the calculation time can be completed within 1 s. The entire detection time shall not exceed 61 s. Compared to traditional chromatography, this method significantly shortens the time. Even compared to the rapid detection method based on spectroscopy, it has advantages. For example, Chakraborty et al. [35] used spectroscopy to detect petroleum hydrocarbons in soil, and the scanning time was at least 90 s. More importantly, the E-nose system is easy to operate, and non-professionals can proficiently use it after simple training. Compared with current rapid detection methods, E-nose systems have significant advantages in detection applicability and accuracy. In terms of detection accuracy, the optimal accuracy for soil condition detection and specific identification of organic pesticides and petroleum hydrocarbons can reach 100%. In terms of practical application and scalability, unlike enzyme inhibition and immunoassay methods [36,37], which are only suitable for detecting organic pesticides in soil, techniques such as X-ray fluorescence spectroscopy [38], are more suitable for detecting petroleum hydrocarbons in soil. The E-nose system developed in this paper can simultaneously detect petroleum hydrocarbons and organic pesticides in soil. This integrated detection system is crucial for improving the efficiency and reliability of large-scale soil pollution detection. On the one hand, compared to multiple independent detection systems, an integrated detection system is easier to integrate into an automation system to obtain comprehensive and integrated soil pollution information. On the other hand, an integrated detection system can effectively reduce equipment procurement and maintenance costs, as well as reduce equipment maintenance and calibration workload, ensuring the accuracy and reliability of the detection system. In addition, by introducing samples such as soil organic matter and moisture content for training, the developed system can also expand functions such as soil fertility and moisture detection, achieving a comprehensive evaluation of soil.

## 4. Conclusions

In this paper, we proposed a rapid and simultaneous detection method of petroleum hydrocarbons and organic pesticides in soil based on E-nose technology. The total volume of the E-nose system developed is 8372 cm^3^, with a total cost of approximately USD 771, suitable for large-scale rapid detection of soil pollution in the agricultural field. Under the joint work of the sampling module and recognition model, the E-nose system can quickly detect soil conditions with an accuracy of up to 100%. Furthermore, the E-nose system can achieve specific identification of organic pesticide types and petroleum types, providing support for precise soil remediation. Among them, using the Max feature extraction method and SVM classifier, the accuracy of six commonly used organic pesticides and two commonly used petroleum products can reach 100%.

The method proposed in this paper can simultaneously achieve rapid detection of petroleum hydrocarbons and organic pesticides in soil, providing a new approach for large-scale detection of soil pollution. In the future, we will continue to optimize the sampling module and recognition model, further reducing equipment size and development costs. We will also introduce better-performing neural network methods to further improve system performance in the face of more complex environments and more target tasks.

## Figures and Tables

**Table 1:** Specific identification results of petroleum types in soil.

Classifiers	Ave	FFT	IV	Max	PCF	WT	Average
SVM	99.58%	90.42%	99.58%	100.00%	89.17%	99.58%	96.39%
RF	99.17%	99.17%	98.33%	99.58%	98.75%	97.92%	98.82%
Average	99.38%	94.80%	98.96%	99.79%	93.96%	98.75%	97.61%

**Table 2:** Specific identification results of organic pesticides in soil.

Classifiers	Ave	FFT	IV	Max	PCF	WT	Average
SVM	99.58%	96.67%	99.58%	100.00%	97.50%	99.58%	98.82%
RF	99.17%	95.83%	99.17%	98.33%	97.92%	99.17%	98.27%
Average	99.38%	96.25%	99.38%	99.17%	97.71%	99.38%	98.55%

## Data Availability

The datasets are available from the corresponding author upon request.

## Appendix A

In Appendix A, we provide detailed information about the sensors used and the optimization scope of each parameter for machine learning.

**Table A1:** Selected gas sensors for the sampling module.

No.	Sensors	Sensitive Gas	Range (ppm)	Manufactor
1	TGS2612	methane, LP, etc.	500~12,500	Figaro
2	TGS2611	methane, natural gas	500~10,000	Figaro
3	TGS2620	ethanol, organic solvents	50~5000	Figaro
4	TGS2603	trimethylamine, methanethiol, etc.	1~10	Figaro
5	TGS2602	ammonia, hydrogen sulfide, etc.	1~30	Figaro
6	TGS2610	LP, propane, butane	500~10,000	Figaro
7	TGS2600	hydrogen, alcohol, etc.	1~30	Figaro
8	GSBT11	volatile organic gases	10~2000	OGAM
9	MS1100	toluene, formaldehyde, benzene, etc.	1~1000	Ruimeng
10	MP135	hydrogen, alcohol, carbon monoxide, etc.	10~500	Winsen
11	MP901	alcohol, formaldehyde, toluene, etc.	1~50	Winsen
12	MP-9	carbon monoxide, methane	300~10,000	Winsen
13	MP-3B	alcohol	0~500	Winsen
14	MP-4	methane, natural gas, methane	300~10,000	Winsen
15	MP-5	propane	300~10,000	Winsen
16	MP-2	propane, smoke	200~10,000	Winsen
17	MP503	alcohol, smoke, isobutane, formaldehyde	1~1000	Winsen
18	MP801	benzene, toluene, formaldehyde, alcohol	0.5~1000	Winsen
19	MP905	benzene, toluene, formaldehyde, alcohol	0.5~1000	Winsen
20	MP402	methane, natural gas	300~10,000	Winsen
21	WSP1110	nitrogen dioxide	0.1~10	Winsen
22	WSP2110	toluene, formaldehyde, benzene, etc.	1~50	Winsen
23	WSP7110	hydrogen sulfide	0~50	Winsen
24	MP-7	carbon monoxide	50~1000	Winsen
25	MP-702	ammonia	0~100	Winsen
26	TGS2618	butane, LP gas	500~10,000	Figaro

**Table A2:** Machine learning optimization parameters and scope.

Classifier	Parameter	Scope
SVM	Penalty coefficient (C)	0.1~100
	Coefficient of kernel function (gamma)	$10^{-6}\sim 1$
	Kernel function	RBF, linear, polynomial
RF	Decision tree	100~500
